# Discovery of a Novel Polyomavirus in Acute Diarrheal Samples from Children

**DOI:** 10.1371/journal.pone.0049449

**Published:** 2012-11-14

**Authors:** Guixia Yu, Alexander L. Greninger, Pavel Isa, Tung G. Phan, Miguel Angel Martínez, Maria de la Luz Sanchez, Juan Francisco Contreras, José Ignacio Santos-Preciado, Julie Parsonnet, Steve Miller, Joseph L. DeRisi, Eric Delwart, Carlos F. Arias, Charles Y. Chiu

**Affiliations:** 1 Department of Laboratory Medicine, University of California San Francisco, San Francisco, California, United States of America; 2 UCSF-Abbott Viral Diagnostics and Discovery Center, San Francisco, California, United States of America; 3 Department of Biochemistry and Biophysics, University of California San Francisco, San Francisco, California, United States of America; 4 Departamento de Genética del Desarrollo y Fisiología Molecular, Instituto de Biotecnología, Universidad Nacional Autónoma de México, Cuernavaca, Morelos, México; 5 Blood Systems Research Institute, San Francisco, California, United States of America; 6 Division of Infectious Diseases and Geographic Medicine, Department of Medicine, Stanford University, Stanford, California, United States of America; 7 Departamento de Microbiología e Inmunología, Universidad Autónoma de Nuevo León, Monterrey, Nuevo León, México; 8 Unidad de Medicina Experimental, Facultad de Medicina, Universidad Nacional Autónoma de México, México, DF, México; Karolinska Institutet, Sweden

## Abstract

Polyomaviruses are small circular DNA viruses associated with chronic infections and tumors in both human and animal hosts. Using an unbiased deep sequencing approach, we identified a novel, highly divergent polyomavirus, provisionally named MX polyomavirus (MXPyV), in stool samples from children. The ∼5.0 kB viral genome exhibits little overall homology (<46% amino acid identity) to known polyomaviruses, and, due to phylogenetic variation among its individual proteins, cannot be placed in any existing taxonomic group. PCR-based screening detected MXPyV in 28 of 834 (3.4%) fecal samples collected from California, Mexico, and Chile, and 1 of 136 (0.74%) of respiratory samples from Mexico, but not in blood or urine samples from immunocompromised patients. By quantitative PCR, the measured titers of MXPyV in human stool at 10% (weight/volume) were as high as 15,075 copies. No association was found between the presence of MXPyV and diarrhea, although girls were more likely to shed MXPyV in the stool than boys (p = 0.012). In one child, viral shedding was observed in two stools obtained 91 days apart, raising the possibility of chronic infection by MXPyV. A multiple sequence alignment revealed that MXPyV is a closely related variant of the recently reported MWPyV and HPyV10 polyomaviruses. Further studies will be important to determine the association, if any, of MXPyV with disease in humans.

## Introduction

Polyomaviruses are small, circular DNA viruses that can cause persistent infections in both animals and humans, and are also potentially oncogenic [1]. In humans, polyomaviruses are associated with a broad spectrum of diseases ranging from progressive multifocal leukoencephalopathy (PML) (JCV, JC virus) to nephropathy (BKV, BK virus), to Merkel cell cancer (MCV, Merkel cell virus) [2], [3], [4], [5]. Ongoing efforts to identify and characterize novel polyomaviruses are important as they may yield valuable insights into the establishment of latent infections and viral carcinogenesis.

The human polyomaviruses JCV and BKV, initially described in 1971 [6], [7], are closely related to each other genetically and have high seroprevalence rates in adults, exceeding 40% [8], [9]. BKV can establish a chronic infection in the kidneys [10], and causes nephropathy and hemorrhagic cystitis in transplant patients [2], although it can also be detected in urine from healthy individuals [8]. JCV also latently infects the kidneys [11], but in immunocompromised individuals, especially in patients with HIV, can invade the central nervous system and cause PML, a life-threatening demyelinating illness associated with headaches, memory loss, and neurological deficits [4]. Up until 2007, the only two polyomaviruses known to infect humans were JCV and BKV, but recent advances in sequencing technologies have since led to the discovery of many additional human polyomaviruses. The WU and KI polyomaviruses were initially described in 2007 in children with acute respiratory illness [12], [13], but the exact pathogenic role of these viruses in respiratory disease remains controversial [14]. These viruses have been found to infect the respiratory tract of up to 7% of children [12], [13], [15], [16], [17], [18], [19], [20], with or without respiratory symptoms, and, like BCV and JCV, seroprevalence rates in both children and adult populations are high [8], [9]. MCV was first described in 2008 in association with a rare but aggressive type of skin cancer called Merkel cell carcinoma (MCC) [3]. In tumor cells, MCV integrates into the host genome and is unable to replicate due to truncation mutations in the viral T antigen [21]. The direct etiologic role of MCV in oncogenesis was demonstrated by cell death and regression of MCC tumors upon knockdown of the viral T antigen [5]. Since the discovery of MCV, three additional human polyomaviruses infecting skin, HPyV6, HPyV7, and TSV (trichodysplasia spinulosa-associated polyomavirus) [22], [23], [24], and a ninth polyomavirus from the blood of immunosuppressed patients, HPyV9, were discovered [25]. Most recently, new polyomaviruses MWPyV and HPyV10 have been detected in human stool specimens [26] and in condyloma (wart) specimens from a patient with WHIM (warts, hypogammaglobulinemia, infections, and myelokathexis) syndrome [27], respectively.

Unbiased DNA sequencing is rapidly becoming the method of choice for pathogen discovery, as high-throughput or “deep” sequencing of clinical samples facilitates the identification of novel, highly divergent pathogens that would elude detection by conventional PCR assays [28], [29]. Previously, we have shown that by shotgun sequencing as few as 1 million reads per clinical sample, sensitivities of detection comparable to PCR (<100 copies per mL) can be achieved for both known and candidate novel viruses [30]. Here we describe the identification and molecular characterization of a new human polyomavirus, provisionally named MX polyomavirus (MXPyV), in diarrheal stool collected from a child in Mexico. Subsequent PCR-based screening of stool samples reveals that the MXPyV has a broad geographic distribution and that persistent shedding of the virus may occur in infected individuals.

## Methods

### Stool sample collection, nucleic acid extraction, and Illumina deep sequencing

Anonymized samples were collected from 96 children with acute diarrheal disease from 3 different states in Mexico between 2008–2009. Diarrhea was defined as three or more loose or liquid stools per day, and samples were taken from children prior to treatment with rehydration and antibiotics (if indicated). Viral particles were purified from stool samples by generating a suspension consisting of 1 mL phosphate-buffered saline, 0.1 g of glass beads, 100 µL of chloroform, and 0.2 g of feces, shaking×5 min using a mechanical shaker, spinning×20 min at 1,000 *g* in a centrifuge, and recovering the aqueous supernatant. 500 µL of supernatant were then passed through a 0.45 µm filter and treated with a cocktail of nucleases (Turbo DNAse, Ambion and RNAseA, Invitrogen) prior to nucleic acid extraction using the PureLink 96 Viral RNA/DNA Kit (Invitrogen). Sample cDNA libraries were prepared from extracted nucleic acid using a random PCR amplification method, separately barcoded, and sequenced on an Illumina HiSeq 2000 as previously described [30], [31]. Raw Illumina sequences consisting of 75 base pair (bp) paired-end reads were filtered to exclude low-complexity, homopolymeric, and low-quality sequences, and then processed through an automated pipeline for pathogen identification as previously described [30]. Sequences corresponding to MXPyV were identified on the basis of viral BlastX homology at a threshold E-score cutoff of 10^−5^.

### PCR for genome recovery

**Figure 1:**
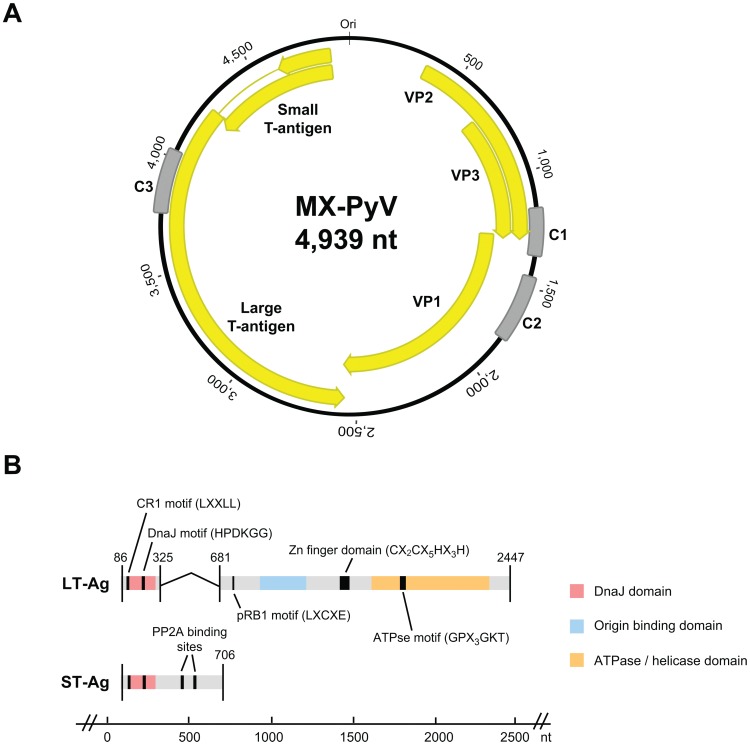
Genome organization of MXPyV. The 4,939-nt circular genome of MXPyV (**A**) contains putative coding regions for VP1, VP2, VP3, ST-Ag, and LT-Ag (yellow arrows). C1, C2, and C3 (gray) denote *de novo* assembled contigs from deep sequencing data. (**B**) Domains and binding motifs present in the spliced LT-Ag and ST-Ag of MXPyV.

Three contigs (contiguous sequences) were assembled from deep sequencing reads bearing homology to polyomaviruses by viral BlastX alignment (marked “C1”, “C2”, and “C3” in Fig. 1). To bridge these contigs, long-range PCR was performed using primers directed outward from the assembled contigs and the PrimeStar GXL DNA Polymerase kit (Takara Bio) according to the manufacturer's instructions. Overlapping PCR products were cloned and sequenced in order to obtain a consensus sequence for the complete MXPyV genome with at least 3× redundancy. Putative open reading frames were identified using Geneious software [32].

### Phylogenetic analysis

**Figure 2:**
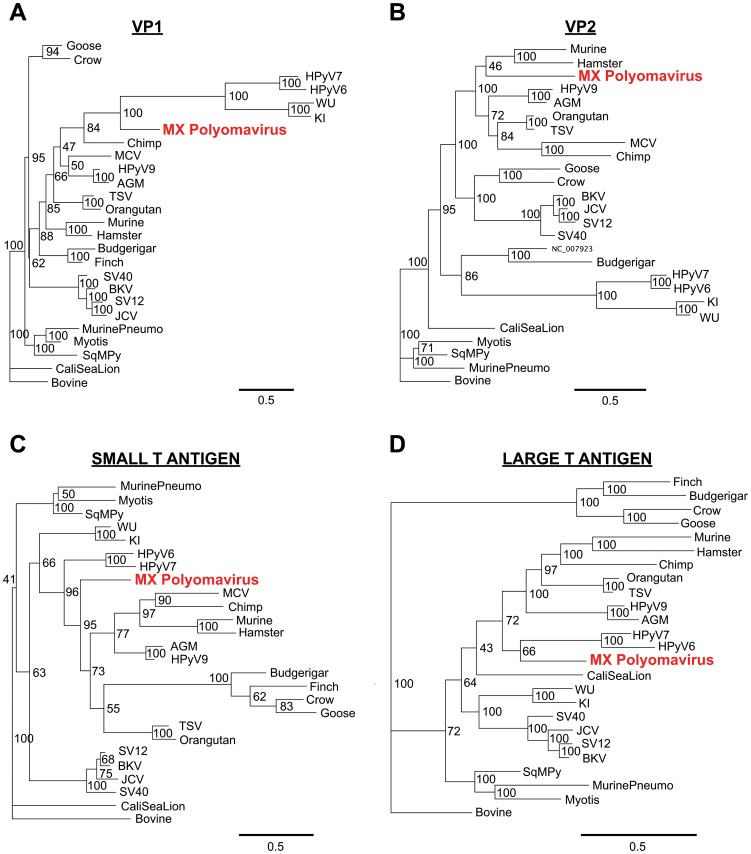
Amino acid phylogenetic analysis of MXPyV relative to other polyomaviruses. (**A**) VP1, (**B**) VP2, (**C**) ST-Ag, (**D**) LT-Ag. Bayesian support levels are indicated at each branching point. Abbreviations: AGM, African green monkey; SV40, simian virus 40; SV12, simian virus 12; SqMPy, squirrel monkey; CaliSeaLion, California sea lion. Other abbreviations and GenBank accession numbers are described in the text. Note that Merkel cell virus (MCV) is not included in the LT-Ag phylogeny due to the presence of truncation mutations.

Whole-genome sequences corresponding to all known animal and human polyomaviruses, with the exception of the recently discovered MWPyV and HPyV10 viruses [26], [27], were downloaded from GenBank. Multiple sequence alignments of MXPyV viral proteins relative to corresponding proteins from other polyomaviruses were performed using MAFFT (v6.0) with the E-INS-i option and at default settings [33]. Overall pairwise amino acid identities of MXPyV relative to other polyomaviruses were calculated by concatenating the VP1, VP2, and T-antigen protein sequences and running MAFFT. To generate the phylogenetic trees, Bayesian tree topologies were calculated using MrBayes V3.2 software (5,500 sampled trees; 500 trees discarded as burn-in for VP1, VP2, and small T antigen; 20,000 sampled trees; 10,000 trees discarded as burn-in for large T antigen needed to achieve convergence) [34]. Bovine polyomavirus (Fig. 2, “Bovine”) was selected as an outgroup. Convergence was confirmed by the PSRF statistic in MrBayes [35]. Trees were visualized using Geneious software [32]. Multiple whole-genome sequence alignments of MXPyV, HPyV10, and MWPyV were performed using Geneious software [32].

### PCR-based screening for MXPyV

A real-time quantitative RT-PCR (qRT-PCR) assay was designed for detection of MXPyV from the VP1 gene, as were two secondary conventional RT-PCR assays from another region of the VP1 gene and the large T-antigen. A reverse transcription step was included for all of the assays in order to enable detection of MXPyV viral mRNA in addition to genomic DNA. To investigate the relative contribution of MXPyV mRNA to viral detection and assess titers of genomic MXPyV, we also performed real-time qPCR on samples found to be MXPyV-positive by qRT-PCR. A standard curve was calculated from 3 PCR replicates at 8 serial log dilutions of a quantified 137-bp MXPyV PCR amplicon (Fig. S1). Assays were performed with the Qiagen One-Step RT-PCR kit using 13.5 µL H_2_O, 5 µL 5× buffer, 1 µL dNTP, 1 µL RT/Taq mix, 1.5 µL of forward and reverse 10 µM primers, 0.5 µL of 2.5× SybrGreen (for the real-time assay), and 2 µL of extracted nucleic acid. MXPyV primers for the PCR-based assays are listed in Table S1. A sample was considered positive for MXPyV if confirmed by Sanger sequencing and at least two of the three PCR-based assays were positive.

### Pan-viral microarray (ViroChip) analysis of MXPyV-positive samples from Mexico

Sufficient material was available from the stool samples from Mexico to test the 12 MXPyV-positive samples for co-infections by pan-viral microarray (ViroChip) and specific PCR analysis for diarrheal viruses. ViroChip analysis was performed as previously described [30], [31]. Briefly, RNA was reverse-transcribed to cDNA using random primers (5′-GTTCCCACTGGAGGATA(N_9_)-3′) and second-strand synthesis was performed using Sequenase. Samples were labeled with Cy3 fluorescent dye, normalized to 10 pmol of incorporated dye, and hybridized overnight to the ViroChip microarray for 16 hr at 65°C. The current 8×60 k version 5.0 (v5.0) ViroChip microarrays used in this study (GEO accession number GPL15905) are manufactured commercially on an Agilent platform (Agilent Technologies), and contain 19,058 70mer oligonucleotide probes representing all viral species in GenBank. Microarrays were scanned at 2 µm resolution on an Agilent DNA Microarray Scanner. Microarray hybridization patterns were interpreted using cluster and single oligonucleotide Z-score analysis as previously described [30], [31], [36], [37]. Samples were declared positive for a diarrheal virus by microarray if positive by both cluster and Z-score analysis.

### Diarrheal viral PCR analysis of MXPyV-positive samples from Mexico

PCR for 5 diarrheal viruses (calicivirus, astrovirus, adenovirus, rotavirus, and enterovirus) was performed using randomly amplified cDNA as a template. Primer pairs are listed in Table S1. All PCR assays were run in a total of 20 µL with 1× PCR buffer, 2 mM MgCl_2_, 0.3 mM dNTP, 10 pmol of each primer, and 1 unit of Taq DNA Polymerase (Invitrogen). Calicivirus, rotavirus, and enterovirus PCRs were run at 94°C×2 min; 35 cycles of 94°C for 30 s, 50°C for 30 s, 72°C for 1 min; and extension at 72°C for 5 min. Adenovirus and astrovirus PCRs were run at 94°C×2 min; 35 cycles of 94°C for 30 s, 55°C for 30 s, 72°C for 1 min; and final extension at 72°C×5 min. Products were visualized on a 1.5% agarose gel stained with ethidium bromide.

### Ethics Statement

Stool and respiratory samples from Mexico, Chile, and the United States were analyzed anonymously. The Institutional Review Boards (IRBs) of the Instituto de Biotecnología, Universidad Nacional Autónoma de México and University of California, San Francisco (UCSF) waived the need for written informed consent as the anonymized, non-identifiable samples were deemed not to constitute human subjects research. Written informed consent was obtained for all participants in the Stanford SIFT (Stanford Infection and Familial Transmission) study for collection of clinical and demographic data and analysis of their samples. For children enrolled in the SIFT study, written informed consent was obtained on their behalf from parents, guardians or immediate next-or-kin. All samples used in the study were collected and analyzed under protocols approved by the IRBs of the Instituto de Biotecnología, Universidad Nacional Autónoma de México, Stanford University, and University of California, San Francisco (UCSF).

### Prevalence study populations

#### Mexico

Stool samples from 96 children with diarrheal disease (including the initial MXPyV-positive case identified) were extracted and tested for MXPyV by PCR. Nasal washes from 136 hospitalized children with pneumonia collected from 2010–2012 were extracted using the PureLink 96 Viral RNA/DNA Kit (Invitrogen) and tested for MXPyV.

#### California (SIFT Study)

The stool samples corresponding to the SIFT (Stanford Infection and Familial Transmission) study have been described previously [38]. Briefly, 553 stool samples from 406 individuals, nearly all children, with or without symptoms of gastroenteritis, were available for study. Stool samples were collected around the time of an initial gastroenteritis episode, and individuals were surveyed for the presence or absence of diarrhea, vomiting, or both within the prior 2 weeks. Additional stool samples were also occasionally collected 3 months after the initial episode. Stool was suspended in 2 mL of PBS at 10% weight per volume and the PureLink 96 Viral RNA/DNA Kit (Invitrogen) was used to extract nucleic acid for MXPyV testing.

#### Chile

192 samples (96 from children with diarrhea and 96 from age-/sex- matched controls) collected between 2009–2011 from Chile were available for testing. Viral particles were enriched by filtration and nuclease treatment prior to nucleic acid extraction using the QIAAMP Viral Ultrasens Kit (Qiagen).

#### California (UCSF Study)

193 plasma samples from solid organ and bone marrow transplant recipients at UCSF sent in 2012 for cytomegalovirus (CMV) testing, with 31 (16%) samples positive for CMV, and 287 plasma/urine samples from predominantly renal transplant recipients sent in 2012 for BKV testing, with 162 (56%) samples positive for BKV, were tested for MXPyV. Viral DNA extractions were performed using the automated Qiagen EZ1 instrument (Qiagen) according to the manufacturer's protocol.

### Nucleotide sequence accession numbers

The annotated, complete genome of MXPyV has been submitted to GenBank (accession number JX259273). Deep sequencing reads corresponding to the diarrheal stool library from which MXPyV was identified have been submitted to the NCBI Sequence Read Archive (accession number SRA056896). All ViroChip microarrays used in this study have been deposited in the NCBI GEO database (accession numbers GSE40008; GSM983236–GSM983247). Accession numbers for the animal and human polyomaviruses used in the phylogenetic analysis are listed as follows: NC_015150, NC_014743, NC_014407, NC_014406, NC_014361, NC_013796, NC_013439, NC_012122, NC_011310, NC_010277, NC_009951, NC_009539, NC_009238, NC_007923, NC_007922, NC_004800, NC_004764, NC_004763, NC_001699, NC_001669, NC_001663, NC_001538, NC_001515, NC_001505, and NC_001442.

## Results

### Discovery and Whole-Genome Sequencing of MXPyV

Eighty stool samples selected from an ongoing investigation of pediatric gastroenteritis from Mexico were analyzed by unbiased Illumina paired-end sequencing. Samples were individually barcoded and sequenced in pools containing 16 samples each. Each pool was subjected to an automated viral discovery pipeline using GenBank database searches and categorized into human, bacterial, phage, unknown, and viral sequences [30]. In one pool consisting of 79,013,460 paired-end sequences, three 100-bp reads, all derived from a single barcoded sample from a 2-year child with diarrhea, were found to have amino acid homology to polyomaviruses by BLASTx. These 3 reads and their corresponding mate pairs were aligned using BLASTn at a E-score cutoff of 10^−10^ to the full deep sequencing dataset corresponding to the barcoded sample (17,981,772 reads) and the resulting identified read pairs assembled to generate 3 contigs (contiguous sequences) 192, 275, and 261 bp in length (Fig. 1, “C1”, “C2”, and “C3”). The closest protein hits to the translated C1, C2, or C3 contigs in the GenBank viral database included VP3 from orangutan polyomavirus (GenBank CAX87756, E-score = 9×10^−11^, 81% identity), VP1 from TSV (GenBank YP_003800006, E-score = 7×10^−30^, 52% identity), and the large T antigen from orangutan polyomavirus (GenBank CAX87759, E-score = 1×10^−25^, 61% identity), respectively. Using long-range PCR with primers directed outward from each of the 3 contigs, the entire genome of the novel polyomavirus was then cloned and sequenced from three overlapping fragments by long-range PCR.

### Genomic organization and phylogenetic analysis

**Figure 3:**
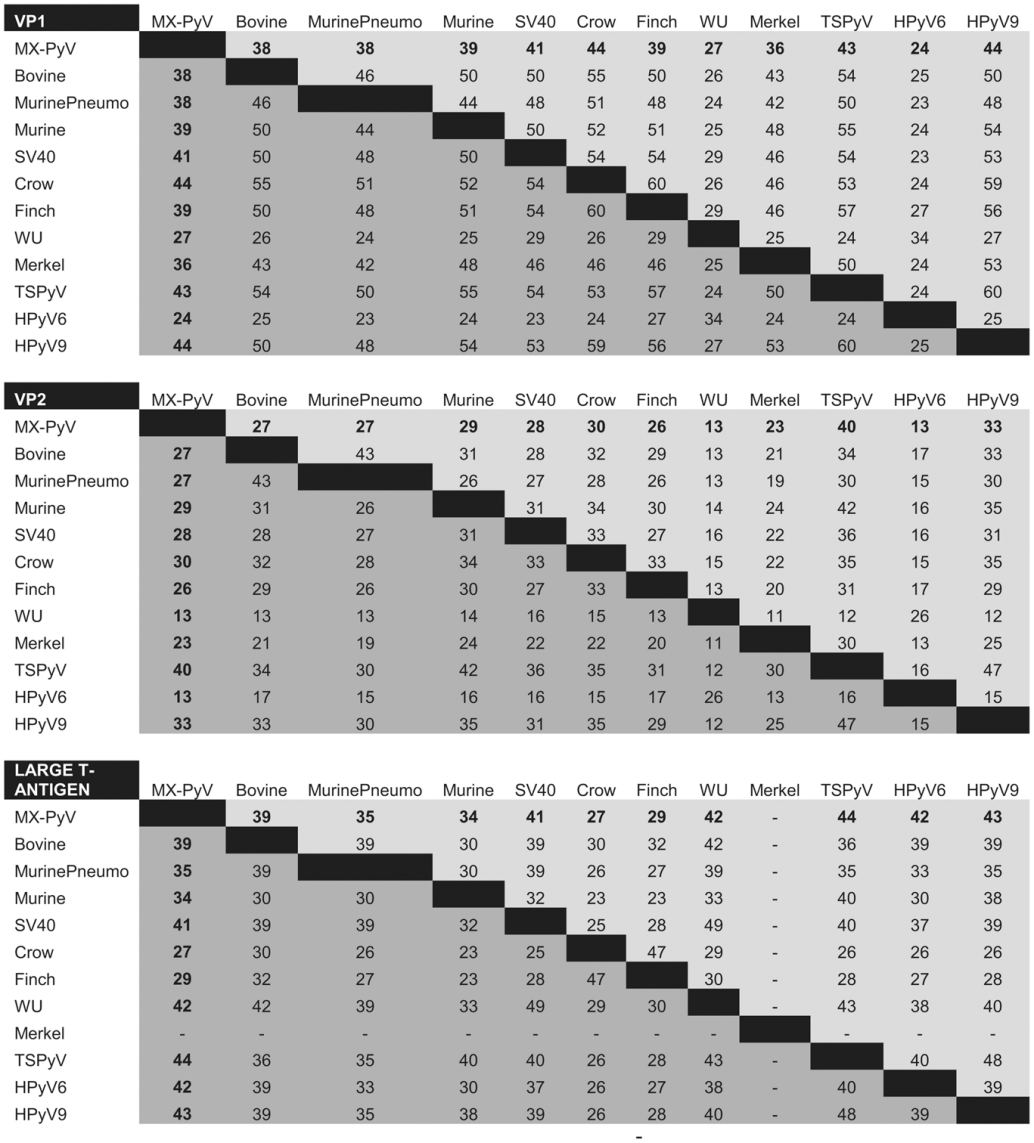
Amino acid identities of the VP1, VP2, small T-antigen, large T-antigen of MXPyV relative to that of other polyomaviruses.

The genome of MXPyV is circular and 4,939 nt in length (accession number JX259273), encoding predicted full-length open reading frames for all of the major polyomavirus proteins (Fig. 1A). The organization is typical for a member of the *Polyomaviridae* family with an early region consisting of regulatory small-T (ST-Ag) and large-T antigens (LT-Ag) and a late region coding for the VP1, VP2, and VP3 structural proteins. Phylogenetic analysis of the VP1, VP2, ST-Ag, and LT-Ag proteins of MXPyV revealed that the taxonomic placement of MXPyV varies from protein to protein (Fig. 2). In VP1 and the large T-antigen, MXPyV shares the most homology with the recently described new human polyomaviruses (HPyV6, HPyV7, WU, and KI), whereas in VP2 or the small T-antigen, MXPyV clusters with the rodent polyomaviruses or forms an independent phylogenetic branch, respectively. Both the regulatory and structural proteins of MXPyV differ substantially in amino acid sequence from those of other polyomaviruses, with identities ranging from 13–44% (Fig. 3).

### Regulatory region

Situated between the early and late regions of polyomaviruses is a non-coding regulatory region which contains the origin of replication as well as transcriptional promoters/enhancers. Typical of nearly all polyomaviruses, the regulatory region of MXPyV was found to contain an AT-rich region on the late side of the putative replication origin (nt 26–57). However, only three T antigen-binding sites, defined by the conserved pentameric GAGGC sequence, were identified in the regulatory region, unlike most polyomaviruses, which contain four to seven such sites. Two of the three T-antigen binding sites in the MXPyV regulatory region were found to combine to form a pentanucleotide palindrome (GAGGCN_4_GCCTC), a feature found in most polyomaviruses. Among human polyomaviruses 1 through 9, only HPyV6 (n = 2) and HPyV7 (n = 1) have fewer T-antigen binding sites than MXPyV.

### Early Region

As typical for polyomaviruses, the LT-Ag of MXPyV is spliced. The donor and acceptor splice site for the LT-Ag of MXPyV were determined based on splice consensus sequences and alignment with the LT-Ag of other polyomaviruses (Fig. 1A). The T-antigen locus of MXPyV contains features conserved with other polyomavirus T antigens, including CR1 (LXXLL), DnaJ (HPDKGG), a pRB1-binding motif (LXCXE), two PP2A binding sites (CXCX_2_C), a zinc finger domain (CX_2_CX_5_HX_3_H), and a helicase/adenosine triphosphatase (ATPse) domain (GPX_3_GKT) (Fig. 1B). The nuclear localization signal and host range domain, though present in SV40, BK, and JC virus [39], [40], [41], [42], [43], do not appear to be conserved in MXPyV.

### Late Region

MXPyV retains the core features common to all known polyomaviruses in the late region, including putative open reading frames for the VP1, VP2, and VP3 capsid proteins, encoding of VP3 in the same ORF as VP2 by use of an internal start codon, and an overlap between VP1 and VP3. Unlike BKV, JCV, SV40, and SV12, there is no ORF for an agnoprotein upstream of the VP2 gene.

### Prevalence of MX polyomavirus in clinical samples

We designed real-time quantitative RT-PCR and PCR assays targeting the VP1 gene to investigate the prevalence of MX polyomavirus in clinical samples (Tables 1 and 2). The inclusion of the reverse transcriptase step greatly improved the sensitivity of detection of MXPyV (Table 2), presumably by enhancing detection of viral mRNA transcripts in infected host cells. RT-PCR/PCR results were confirmed by visualization of an expected-size band on gel electrophoresis, melting curve analysis, and sequencing. All positive results were also independently confirmed using two additional conventional RT-PCR assays targeting the LT-Ag gene and a different region of the VP1 gene. MXPyV was detected in stool samples from children with or without diarrhea on two continents, with prevalence rates of 12.5% (12 of 96) in Mexico, 3.3% (18 of 546) in California, and 4.2% (4 of 96) in Chile. Sequence variation within the 138 nt fragment varied from 0.0–4.3% (data not shown). Analysis of MXPyV-positive stools from Mexico using the ViroChip pan-viral microarray and diarrheal virus PCR identified known pathogenic diarrheal viruses in 50% (6 of 12) samples (Table S2). Among the MXPyV-positive samples from California for which clinical and demographic data were available, no association was noted between diarrhea and MXPyV infection (Table 3). Interestingly, a child from California was found to be MXPyV-positive both at the time of an acute gastroenteritis episode and 3 months later, suggesting that persistent viral shedding of MXPyV in stool may occur (Table 4). In addition, girls overall were found to be more likely infected by MXPyV than boys (p = 0.012) (Table 4). Given the known association of BK and JC virus with disease or asymptomatic shedding in immunocompromised individuals, we also screened for MXPyV in 480 plasma and urine samples from transplant patients at a single hospital in California, with all samples testing negative. Furthermore, 136 respiratory samples from Mexico from hospitalized children with pneumonia were screened, with only one sample (0.74%) confirmed positive for MXPyV infection (Table 1). This sample corresponded to a child with pneumonia who was also found to be co-infected with a rhinovirus/enterovirus by RT-PCR.

**Table 1:** Results from MXPyV screening of clinical samples by RT-PCR.

Geographic Source	Sample type	Subjects	# of Samples Tested	# of MXPyV-Positive Samples (%)
Mexico	stool	children with diarrhea	96	12 (12.5%)*
Mexico	nasal washes	children with respiratory infection	136	1 (0.74%)
California (SIFT**)	stool	children with or without diarrhea	546	18 (3.3%)
California (UCSF)	plasma	transplant recipients	193	0 (0.0%)
California (UCSF)	plasma/urine	transplant recipients	287	0 (0.0%)
Chile	stool	children with diarrhea	96	0 (0.0%)
Chile	stool	age-/sex-matched controls (children without diarrhea)	96	4 (4.2%)

* includes initial MXPyV-positive sample identified by deep sequencing.

** Stanford Infection and Familial Transmission Study.

**Table 2:** Comparison of quantitative RT-PCR vs. PCR assays for detection of MXPyV and titers of MXPyV in stool.

	qRT-PCR (Ct)	qPCR (Ct)	qPCR (copies/mL)
Mex-1	35.65	38.13	708
Mex-2	30.92	36.40	1,800
Mex-3	33.25	-	-
Mex-4	26.3	33.46	8,791
Mex-5	29.98	37.19	1,176
Mex-6	33.69	-	-
Mex-7	30.03	37.05	1,268
Mex-8	27.3	34.35	5,439
Mex-9	33.1	37.0	1,303
Mex-10	*	*	*
CA-1	30.86	35.01	3,810
CA-2	27.51	-	-
CA-3	31.95	36.72	1,515
CA-4	33.73	-	-
CA-5	32.21	38.40	612
CA-6	35.24	37.05	1,268
CA-7	31.75	35.14	3,552
CA-8	30.74	-	-
CA-9	30.97	39.81	286
CA-10	30.6	-	-
CA-11	26.68	32.46	15,075
CA-12	31.57	-	-
CA-13	38.9	-	-
CA-14	34.4	-	-
CA-15	*	*	*
CA-16	*	*	*
CA-17	*	*	*
CA-18	*	*	*
CA-19	*	*	*
CA-20	*	*	*
Chile-1	33.08	-	-
Chile-2	*	*	*
Chile-3	*	*	*
Chile-4	*	*	*

Abbreviations: Mex, Mexico; CA, California.

-, not detected by qPCR.

* , not tested by PCR because of sample unavailability or because sample was negative by qRT-PCR but positive by other qualitative RT-PCR assays.

**Table 3:** Gastroenteritis symptoms corresponding to MXPyV-positive stool samples compared to uninfected samples in the California SIFT study.

	Symptoms		
Stool Samples (n = 546)	Diarrhea N(%)	Vomiting N(%)	Diarrhea or vomiting
MXPyV-positive (n = 18)	10 (56%)	9 (50%)	11 (61%)
MXPyV-negative (n = 528)	271 (51%)	245 (46%)	317 (60%)
P-value	0.81	0.81	1.0

**Table 4:** Demographics of individuals who provided stool samples from the California SIFT study according to MXPyV positivity.

	At least one positive stool sample	Negative sample(s)**	Total
Total	17* (4%)	389 (97%)	406
Male‡	4 (24%)	211 (54%)	215
Mean age, years	1.76	2.21	2.19
Median age, years	1.59	1.06	1.08
Age range, years	0.34–60.8	0.87–6.01	

* One person provided two samples and 16 provided 1. The individual who provided two samples corresponded to a child who was MXPyV-positive both at the time of an acute diarrheal episode and 3 months later.

** 146 people provided two samples and 243 provided one.

‡ Gender difference significant at p = 0.012 (Fisher's Exact Test).

## Discussion

Here we identified and sequenced the entire genome of a novel, highly divergent polyomavirus by deep sequencing of diarrheal samples. In accordance with the two-letter designations for human polyomaviruses, we have provisionally named this virus MX polyomavirus (MXPyV), after the country from which the initial isolate was identified. The genomic organization and amino acid sequence homology of MXPyV, as well as conservation of known protein motifs in the T-antigen, indicate that this virus is indeed a polyomavirus. MXPyV is broadly distributed and was recovered from diarrheal samples from two continents, as well as from respiratory secretions from a child with pneumonia. In addition, independent MXPyV isolates from different individuals showed sequence variation of 0–4.3%, and the virus was detected in children from birth to 6 years of age.

**Figure 4:**
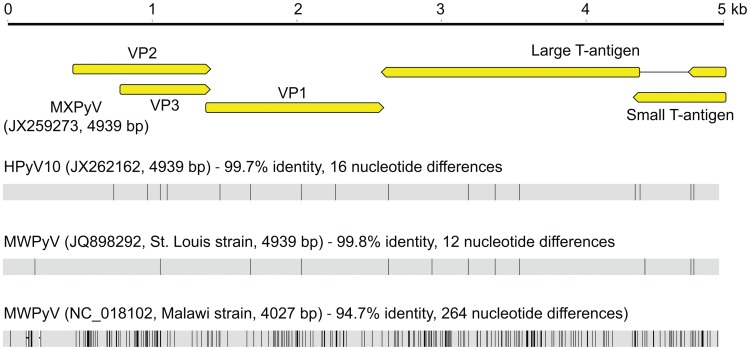
Whole-genome sequence alignment of MXPyV relative to other recently described gut-associated polyomaviruses HPyV10 and MWPyV.

By phylogenetic analysis, MXPyV does not consistently cluster with any other polyomavirus taxonomic group and, indeed, whereas MXPyV ORFs encoding VP1 and the large T-antigen cluster with human polyomaviruses (WU, KI, HPyV6, and HPyV7), the MXPyV ORF encoding VP2 appears to group better with rodent polyomaviruses. In contrast, the small T-antigen of MXPyV does not appear to cluster with any of the known polyomavirus groups. These observations, combined with the low amino acid identity of 13–44% in the proteins of MXPyV relative to those of other polyomaviruses (Fig. 3), suggest that the putative ancestral strain for MXPyV likely diverged early along the evolutionary pathway, and raises the possibility of recombination of polyomavirus genes. Although recombination in polyomaviruses remains controversial, it does appear to occur, at least in JC viruses [44]. No evidence for MXPyV recombination within individual genes was detected by bootscanning analysis (data not shown), but this is to be expected given the high sequence divergence of MXPyV. The whole-genome sequence of MXPyV is nearly identical to that of the recently described gut-associated polyomavirus MWPyV (St. Louis strain) or HPyV10 [26], [27], sharing 99.8% or 99.7% identity, respectively, and thus all 3 viruses are different variants of the same species (Fig. 4). Situated on a highly divergent phylogenetic branch, MXPyV, MWPyV, and HPyV10 likely represent the first members of a new subclade of polyomaviruses.

Detection of MXPyV, as well as closely related strains MWPyV and HPyV10, appears largely confined to the gastrointestinal tract. MXPyV exhibited an overall prevalence of 3.4% in fecal samples collected from California, Mexico, and Chile (Table 1), although one respiratory sample out of 136 (0.74%) also tested positive. SV40, BKV, JCV, and MCV have also been detected in human feces [45], [46], [47], although their primary sites of pathology are elsewhere in the human body, as have polyomaviruses WU and KI [12], [48], [49]. We were unable to detect MXPyV in 480 plasma or urine samples from highly immunocompromised transplant recipients, indicating that these are not reservoir sites for MXPyV infection, as is the case for JC and BK viruses.

No association between MXPyV presence and diarrhea was detected in the California and Chile gastroenteritis studies for which controls were available (Tables 1 and 2). In fact, in the samples from Chile, the trend was reversed, with 4 MXPyV-positive samples among 96 asymptomatic control individuals and no positives among 96 children with diarrhea (Table 1). These findings, however, do not preclude the possibility of MXPyV as an etiologic agent of diarrhea given the fact that a large proportion of infections from diarrheal viruses are asymptomatic [50], [51]. Notably, 6 of 12 MXPyV-positive diarrheal samples from Mexico tested negative by a broad-spectrum viral microarray and specific PCR assays for all known diarrheal viruses (Table S2), suggesting that MXPyV, if human-tropic, may still potentially be a cause of gastroenteritis. Serologic testing before and after diarrheal episodes would be useful in investigating this possibility, as shown previously for a human cardiovirus and klassevirus/salivirus [52], [53].

In the California SIFT study, MXPyV was seen more often in girls than in boys (13 female vs. 4 males, p = 0.012) by RT-PCR (Table 4). Although MXPyV-specific serology is needed for confirmation, this observation is intriguing in light of the fact that apparent gender differences have previously been described in a serological investigation of primary infections by Merkel cell virus (MCV) in childhood [54]. In that study, males showed higher seroconversion and seroprevalence rates to MCV than females. This apparent gender difference was not observed with respect to MCV seroprevalence in adults [9], although gender does appear to dramatically impact incidence and survival rates associated with Merkel cell carcinoma [55], [56]. Whether differences in the age at which MXPyV is acquired, childhood physiology, or viral characteristics play a role in the gender differences observed here is unknown, and merits further investigation.

Although at present we cannot exclude the possibility that MXPyV may be of dietary origin, several lines of evidence indicate that the virus is likely human-tropic. The enhanced sensitivity of RT-PCR over PCR for detection of MXPyV (Table 2) suggests that expressed viral mRNA, presumably present in infected host cells in the feces, is being detected, implying that viral replication occurs in the human gut. In addition, the detection of MXPyV in a child at the time of an acute gastroenteritis episode and 3 months later suggests that, in analogy with other human polyomaviruses [57], chronic infection by MXPyV is possible. The detection of a closely related variant to MXPyV, HPyV10, in tissue from a patient with WHIM syndrome also indicates that MXPyV, MWPyV, and HPyV10 are likely human-tropic viruses (Fig. 4). Viral cultivation or serology will be needed for definitive confirmation that these novel polyomaviruses can cause *bona fide* infections in humans.

## Supporting Information

Figure S1 **Log plot of a standard curve corresponding to a real-time PCR assay for MXPyV.** Each data point is an average of three independent replicates. The standard curve is calculated by logarithmic regression across all 8 data points.
(TIF)

Table S1 **PCR primer sequences used for MXPyV whole-genome assembly, MXPyV screening, and diarrheal virus screening.**
(DOCX)

Table S2 **Other diarrheal viruses found in MXPyV-positive samples (12 of 96, 12.5%) from children in Mexico with acute gastroenteritis.** Abbreviations: TTV, torque teno virus.
(DOCX)
