# Supplementary figures and images for: Discovery of a Novel Polyomavirus in Acute Diarrheal Samples from Children
Source: PLoS One. 2012 Nov 14;7(11):e49449. doi: 10.1371/journal.pone.0049449 (PMC3498111; doi:10.1371/journal.pone.0049449)

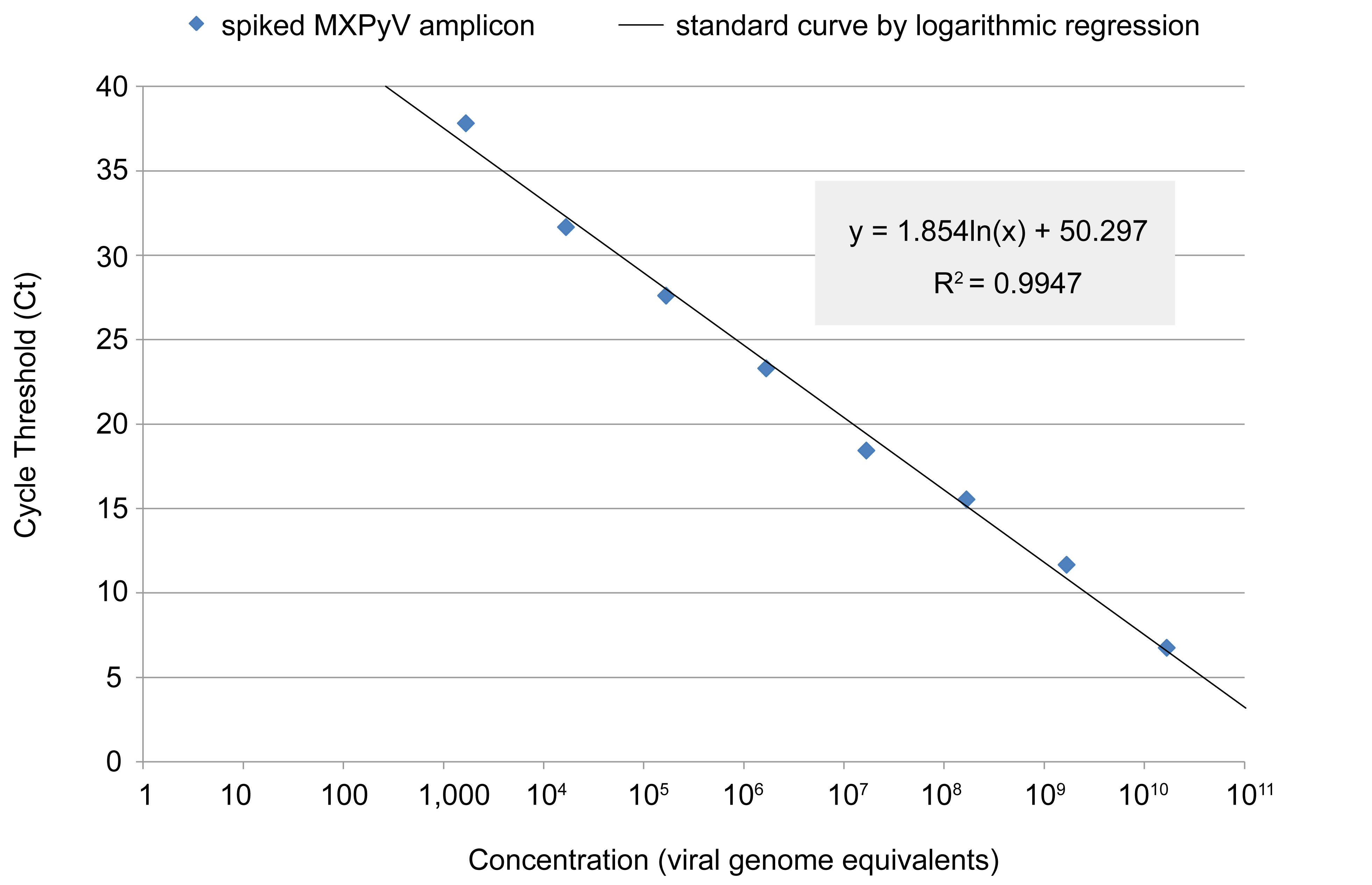

Supplement: Figure S1 — Log plot of a standard curve corresponding to a real-time PCR assay for MXPyV. Each data point is an average of three independent replicates. The standard curve is calculated by logarithmic regression across all 8 data points. (TIF) [file pone.0049449.s001.tif]
