# Supplementary material for: Discovery of a Novel Polyomavirus in Acute Diarrheal Samples from Children
Source: PLoS One. 2012 Nov 14;7(11):e49449. doi: 10.1371/journal.pone.0049449 (PMC3498111; doi:10.1371/journal.pone.0049449)
Supplement: Table S1 — PCR primer sequences used for MXPyV whole-genome assembly, MXPyV screening, and diarrheal virus screening. (DOCX) [file pone.0049449.s002.docx]

**TABLE S1.** PCR primer sequences used for MXPyV whole-genome assembly, MXPyV screening, and diarrheal virus screening.

| Primers for MXPyV Whole-Genome Assembly | |  |  |
| --- | --- | --- | --- |
| Primer | Sequence |  |  |
| MX-VP1-77F | GGTTGAAGAATGACCTCAACTGTC |  |  |
| MX-VP1-231R | GTATATGTGGGAGGCAGTTGTTC |  |  |
| MX-VP3-F | GACAGACTCCTGATTGGATG |  |  |
| MX-largeT-F | CCTGTAGATTTTCCTGAAGTACTC |  |  |
| MX-VP1-1F | CGGACACCACAATGACAGTTGA |  |  |
| MX-VP3-R | GCTTCTGCTCTGGTACAAACAG |  |  |
| MX-largeT-R1 | GCTCACTGTACACAGATTTGAAC |  |  |
| MX-2500-1F | AAGCTACAGACTGGGTCAC |  |  |
| MX-2500-2R | TCTTCTCTGAGCAGTGAC |  |  |
| MX-2500-1R | CTACAGTATTACTGGATG |  |  |
| MX-2500-2F | CTGCTGTTACATATAGCC |  |  |
| MX-2000-2R | GTCCAGAGTTAACCTGTG |  |  |
| MX-2000-3R | CCTGGATATAGACACTTTG |  |  |
| MX-2500-4004 | GTGTCGTCACTTGGCATA |  |  |
| MX-2500-3607 | ATAGTAATAATACCTGGG |  |  |
| MX-smallT-1724 | TTAAAACTGCACCCTGAC |  |  |
|  |  |  |  |
| Primers for MXPyV screening | |  |  |
| MX-Scr-VP1-523F | GAGGCCTGGGCTCCAGATC |  |  |
| MX-Scr-VP1-660R | CCCACACCTCTATCATCCAG |  |  |
| MX-Conf-largeT-1F | CCTGTAGATTTTCCTGAAGTACTC |  |  |
| MX-Conf-LargeT-2R | GCTCACTGTACACAGATTTGAAC |  |  |
| MX-Conf-VP1-661Fdeg | CCCYTGTGTAARGGAGATGGG |  |  |
| MX-Conf-VP1-807Rdeg | AGGRTAAGGATTTYTAACAGCYCTT |  |  |
|  |  |  |  |
| Primers for Diarrheal Virus Screening | |  |  |
| Primer | Sequence | Reference | PMID |
| Calici-F | GATTACTCCARGTGGGAYTCMAC | Farkas et al. (2004) | 15221533 |
| Calici-R | TGACRATKTMATCATCMCCRTA |  |  |
| Adeno-F | GCCGCAGTGGTCTTACATGCACATC | Echavarria et al. (1998) | 9774586 |
| Adeno-R | CAGCACGCCGCGGATGTCAAAGT |  |  |
| Entero- F | CGGCCCCTGAATGCGGCTAA | Rotbart et al. (1990) | 2157735 |
| Entero-R | ATTGTCACCATAAGCAGCC | Benschop et al. (2006) | 16355330 |
| Rota -F | AAGTAGCTGGATTTGATTATTC | Schwarz et al. (2002) | 12270660 |
