# Supplementary material for: Discovery of a Novel Polyomavirus in Acute Diarrheal Samples from Children
Source: PLoS One. 2012 Nov 14;7(11):e49449. doi: 10.1371/journal.pone.0049449 (PMC3498111; doi:10.1371/journal.pone.0049449)
Supplement: Table S2 — Other diarrheal viruses found in MXPyV-positive samples (12 of 96, 12.5%) from children in Mexico with acute gastroenteritis. Abbreviations: TTV, torque teno virus. (DOCX) [file pone.0049449.s003.docx]

| MXPyV-Positive Sample # | Virochip Microarray | PCR | Diarrheal Virus Present? |
| --- | --- | --- | --- |
| 1 | - | - | - |
| 2 | - | - | - |
| 3 | - | - | - |
| 4 | TTV* | - | - |
| 5 | rotavirus A | rotavirus A | + |
| 6 | norovirus | norovirus | + |
| 7 | rotavirus A | rotavirus A | + |
| 8 | rotavirus A | rotavirus A | + |
| 9 | - | - | - |
| 10 | rotavirus A, adenovirus | rotavirus A, adenovirus | + |
| 11 | astrovirus | astrovirus | + |
| 12 | - | - | - |
|  |  |  |  |
| *TTV is considered a non-pathogenic virus | | | |

**Table S2**. Other diarrheal viruses found in MXPyV-positive samples (12 of 96, 12.5%) from children in Mexico with acute gastroenteritis. Abbreviations: TTV, torque teno virus.
